# Rare STAT3 haplotypes cause a predisposition to developing congenital anomalies of the kidney and urinary tract disorder

**DOI:** 10.55730/1300-0144.5911

**Published:** 2024-10-20

**Authors:** Mert POLAT, Feride İffet ŞAHİN, Esra BASKIN, Uğur TOPRAK, Kaan Savaş GÜLLEROĞLU, Mehmet HABERAL, Yunus Kasım TERZİ

**Affiliations:** 1 Department of Medical Genetics, Faculty of Medicine, Başkent University, Ankara, Turkiye; 2 Division of Pediatric Nephrology, Department of Pediatrics, Faculty of Medicine, Başkent University, Ankara, Turkiye; 3 Department of Biostatistics, Faculty of Medicine, Başkent University, Ankara, Turkiye; 4 Division of Transplantation, Department of Surgery, Faculty of Medicine, Başkent University, Ankara, Turkiye

**Keywords:** Congenital anomaly, kidney, urinary tract, CAKUT, STAT3

## Abstract

**Background/aim:**

Congenital anomalies of the kidney and urinary tract (CAKUT) are characterized by renal developmental disorders in the embryonic period. STAT3 is a member of the STAT protein family. The members of this protein family play roles in various cellular mechanisms, such as the early stages of embryonic development, kidney development, and renal diseases. This study aims to determine the frequency of STAT3 rs1053004, rs744166, rs3816769, and rs4796793 polymorphisms in individuals with CAKUT.

**Materials and methods:**

Two of four polymorphisms, rs744166 (c.-1-13666T>C, NM_001369512.1) and rs4796793 ( c.-1915C>G, NM_001369512.1), were analyzed by a polymerase chain reaction (PCR) and the restriction fragment length polymorphism method. Two other polymorphisms, rs1053004 (c.*1671C>T, NM_001369512.1) and rs3816769 (c.273+314A>G, NM_001369512.1), were analyzed using real-time PCR-melting curve analysis.

**Results:**

Our research indicates that individuals with the TT allele for rs1053004 single nucleotide polymorphism have a 1.23 times greater disease risk than those with the CC allele. Those with the CC allele for rs3816769 have a 1.41 times greater risk of disease than those with the TT allele. These findings suggest a potential genetic predisposition to CAKUT. Furthermore, the research identified significant connections between rare haplotypes and CAKUT (p = 0.041). The CCTC haplotype for rs744166, rs4796793, rs1053004, and rs3816769 polymorphism was exclusively present in the CAKUT group, while the CGTT haplotype for the same polymorphisms was only detected in the control group.

**Conclusion:**

The presence of rare haplotypes for the rs1053004, rs3816769, rs4796793, and rs744166 polymorphisms may significantly affect the onset or prevention of CAKUT. These findings could potentially have important clinical implications, providing a deeper understanding of the genetic basis of CAKUT and potentially influencing future diagnostic and treatment strategies.

## 1. Introduction

Renal developmental disorders in the embryonic period characterize congenital anomalies of the kidney and urinary tract (CAKUT). These structural malformations account for approximately 20–30% of all congenital malformations [1,2]. CAKUT comprise problems with urinary tract development that cause several structural malformations. These include renal agenesis, multicystic dysplastic kidney, duplex kidney, renal dysplasia, renal hypoplasia, hydronephrosis, hydroureter, and vesicoureteral reflux [3,4]. CAKUT can be severe, resulting in end-stage kidney disease in childhood, which is clinically significant. The genetics of CAKUT were previously examined in various studies. Thirty-six genes were found to be associated with CAKUT pathogenesis. Nevertheless, mutations in one of these 36 genes are identified in only 20% of patients with CAKUT or extrarenal symptoms. This emphasizes the substantial genetic heterogeneity present in CAKUT [4–7].

The JAK/STAT pathway is critical in the signaling process through cytokine and growth hormone receptors. Activation of this pathway has been linked to several human renal diseases, including diabetic nephropathy and renal fibrosis resulting from unilateral ureteral obstruction [8].

STAT3 is a member of the STAT protein family, which is involved in many cellular processes, including inflammation regulation, immune response, apoptosis, early embryonic development, and cancer [9–10]. The *STAT3* gene is located in the 17q21.2 chromosomal region [9]. It was previously demonstrated that the *STAT3* gene is vital in kidney development and normal functioning. Talbot et al. demonstrated high activity of the *STAT3* gene in renal tubular epithelial cells at postnatal day 7 in mice [10]. In addition, previous research has shown that STAT3 is involved in both tubular epithelial and interstitial cells [11].

The interaction of leukemia inhibitory factor receptor (LIFR) gene signaling and its interaction with the JAK/STAT, MAPK, and PI3K pathways have also been studied in CAKUT patients. A mutation in the *LIFR* gene reduced STAT3 phosphorylation [12], which indicates a correlation between STAT3 phosphorylation and kidney development. These findings imply that both transcriptional and posttranslational control of STAT3 is essential in kidney development and function.

In this study, we analyzed the frequencies of four different single nucleotide polymorphisms (SNPs) of the *STAT3* gene that are important in controlling STAT3 expression in patients with CAKUT. These polymorphisms were selected due to their effects on *STAT3* gene expression and its posttranscriptional and posttranslational outcomes. The analyzed polymorphisms are located in critical regions that control gene expression, splicing, and transcript stability. The rs1053004 (c.*1671C>T, NM_001369512.1) polymorphism is in the 3’ untranslated region (UTR) of the *STAT3* gene and functions as a miRNA binding region [13]. The allele frequencies of the rs1053004 polymorphism across various genetic ancestry groups, as per the Genome Aggregation Database (gnomAD) v4.1.0 [14], are as follows: African/African American: 0.1230, Admixed American: 0.6960, European (non-Finnish): 0.6395, Ashkenazi Jewish: 0.6275, Middle Eastern: 0.6137, East Asian: 0.6012, and European (Finnish): 0.5950. The rs744166 (c.-1-13666T>C, NM_001369512.1) polymorphism is in the intronic region between exon one and two, potentially affecting STAT3 expression and splicing. The allele frequencies of the rs744166 polymorphism, according to the gnomAD v4.1.0 [14], are as follows: African/African American: 0.6782, Admixed American: 0.3096, European (non-Finnish): 0.4118, Ashkenazi Jewish: 0.4083, Middle Eastern: 0.3878, East Asian: 0.4157, and European (Finnish): 0.4220. The rs4796793 (c.-1915C>G, NM_001369512.1) polymorphism is in the 5’ UTR, potentially affecting the transcript’s stability. The allele frequencies of the rs4796793 polymorphism, according to the gnomAD v4.1.0 [14], are the following: African/African American: 0.5049, Admixed American: 0.7980, European (non-Finnish): 0.7458, Ashkenazi Jewish: 0.6916, Middle Eastern: 0.7313, East Asian: 0.6036, and European (Finnish): 0.7340. Finally, the rs3816769 (c.273+314A>G, NM_001369512.1) polymorphism has an intronic location and has been reported to trigger overexpression of the *STAT3* gene [16–19]. The allele frequencies of the rs3816769 polymorphism, according to the gnomAD v4.1.0 [14], are as follows: African/African American: 0.3734, Admixed American: 0.2461, European (non-Finnish): 0.3455, Ashkenazi Jewish: 0.3779, Middle Eastern: 0.3469, East Asian: 0.4175, and European (Finnish): 0.3849. The impact of the STAT3 gene on organ development is well-documented. However, there is a research gap in potential correlations between four genetic variations (rs1053004, rs744166, rs4796793, and rs3816769) in the STAT3 gene and kidney development, specifically within the CAKUT phenotype. This study is the first investigation of the influence of these genetic variations within the CAKUT phenotype.

## 2. Materials and methods

### 2.1. Patients

One hundred forty-five patients with CAKUT with a mean age of 7 years two months (±5.7) and 128 control individuals with a mean age of 7 years ten months (±5.4) were included in the study. The individuals in the control group were selected among healthy children who consulted with the Department of Pediatrics for routine checkups. The clinical examination of the individuals in the control group showed no signs related to CAKUT. Informed consent was obtained from all patients and their parents. CAKUT diagnoses were established at Başkent University’s Division of Pediatric Nephrology Department of Pediatrics. Başkent University’s local ethics committee approved the study, and the research team received support from the institutional review board of Başkent University’s Research Fund (Project no: KA18/189).

### 2.2. Genotyping

Genomic DNA was extracted from peripheral blood lymphocytes from all patients and control individuals. Two of the four polymorphisms, rs744166 (c.-1-13666T>C) and rs4796793 (c.-1915C>G), were analyzed using a polymerase chain reaction and restriction fragment length polymorphism method (PCR-RFLP). Two other polymorphisms, rs1053004 (c.*1671C>T) and rs3816769 (c.273+314A>G/T), were analyzed by real-time PCR-melting curve analysis.

Primer sequences for the PCR-RFLP analysis are given in Table 1. Thermal cycling for rs744166 started with an initial denaturation step at 94 °C for 5 min, followed by 40 cycles at 94 °C for 30 s, 57 °C for 30 s, and 68 °C for 30 s, with a final step at 68 °C for 30 s. Thermal cycling conditions for rs4796793 consisted of an initial denaturation step at 94 °C for 5 m, followed by 40 cycles at 94 °C for 30 s, 60 °C for 30 s, 68 °C for 30 s, and a final step at 68 °C for 30 s. The final concentrations of the PCR contents were 0.4 μM of each primer, 0.1 mM of dNTP (Thermo Fisher Scientific, Waltham, MA, USA), and 0.03 U/μL of Hot Start Taq DNA polymerase (NEB). The resulting 502 bp and 329 bp amplicons were then digested with the restriction enzymes HpyF10VI (MwoI) (Thermo Fisher Scientific, Waltham, MA, USA) and AluI (Thermo Fisher, Waltham, MA, USA) to detect rs4796793 and rs744166 SNPs, respectively.

Genotypes for rs1053004 and rs3816769 SNPs were determined using a real-time PCR-melting curve analysis kit, following the manufacturer’s instructions (Tib Molbiol LightMix Kit, Catalog Number: TIB RS1053004 and TIB RS3816769). The analysis started with a preincubation step at 95 °C for 10 m, followed by 45 cycles at 95 °C for 10 s, 60 °C for 10 s, and, finally, 72 °C for 15 s. A melting curve analysis was then conducted with an initial denaturation at 95 °C for 30 s, followed by hybridization and a stepwise temperature increment from 40 °C to 75 °C at a ramp rate of 1.5 °C/s continuously.

### 2.3. Statistical analysis

SPSS 25 and snpStats library in the Bioconductor V3.14 R V.4.1.1 statistical programming language was used to analyze the collected data. Pearson’s chi-squared test and the Fisher–Freeman–Halton exact test were conducted to identify whether there were any significant differences between the groups. The Kruskal–Wallis test was performed to determine whether significant differences existed between allele distributions. The demographic parameters were identified with descriptive statistics and presented as median ± standard deviation (min–max) separately for the patient and control groups. Odds ratio (OR) analysis is a statistical technique used to measure the strength and direction of association between variables [15]. Logistic regression analysis was conducted to calculate OR with corresponding confidence intervals. The two-stage iterative method via the expectation–maximization algorithm was employed for the haplotype analysis under Hardy–Weinberg equilibrium (HWE). The Hardy-Weinberg principle in population genetics posits that in the absence of disruptive evolutionary factors, allele, and genotype frequencies remain constant across generations. This principle serves as a foundational concept for understanding genetic equilibrium within a population. These analyses were conducted with a 95% confidence level and 80% power conditions, with statistical significance determined at p < 0.05.

## 3. Results

The demographic and clinical characteristics of the patients and control individuals are given in Table 2. A total of 145 patients and 128 control individuals were included in the study.

**Figure 1:**
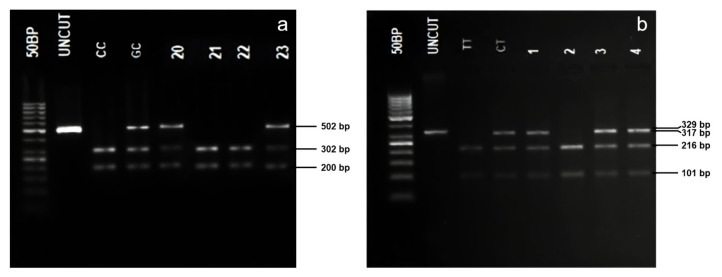
Analyses of the rs744166 (A) and rs4796793 (B) SNPs by PCR-RFLP method. a.) 50 bp is the molecular marker, UNCUT is the undigested PCR product (502 bp), CC genotype (302, 200 bp), GC genotype (502, 302, 200 bp), and 21–23 are patient samples; b.) 50 bp is the molecular marker, UNCUT is the undigested PCR product (329 bp), TT genotype (216,101, 8, and 4 bp), CT (317,216,101, 8, and 4 bp), and 1–4 are patient samples.

Two polymorphisms (rs744166 and rs4796793) were examined using PCR-RFLP analysis. The enzymatic digestion of rs744166 (c.-1-13666T>C) generated four fragments (216, 101, 8, and 4 bp) in the TT genotype and three fragments (317, 8, and 4 bp) in the CC genotype. The enzymatic digestion of rs4796793 generated two fragments (302 and 200 bp) in the CC genotype and only one (undigested) in the GG genotype. Gel images of the digested products for rs4796793 and rs744166 SNPs are given in Figures 1a and 1b. Genotypes for rs1053004 and rs3816769 SNPs were determined with real-time PCR-melting curve analysis.

Genotype frequencies and OR of the examined SNPs are given in Table 3. The results reveal that the CAKUT patients exhibited higher frequencies of the CT-TT genotype in rs1053004, TC-CC genotype in rs3816769, GC-GG genotype in rs4796793, and TC-CC genotype in rs744166 compared to the healthy controls. In addition, we determined that individuals with the TT allele for rs1053004 SNP have a 1.23 times greater risk of disease than those with the CC allele, and the CC allele for rs3816769 has a 1.41 times greater risk of disease than those with the TT allele.

We analyzed the genotype frequencies in the patient and control groups to determine whether they were consistent with HWE (Table 4). The results indicate that the data in our patient group showed compliance with HWE.

**Figure 2:**
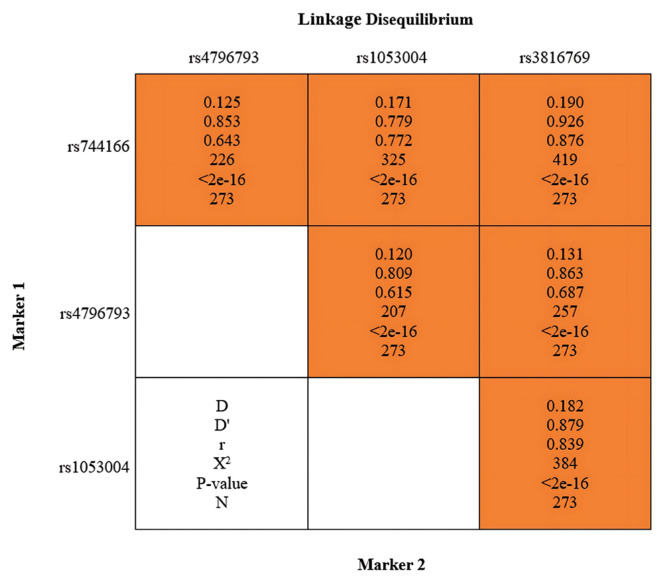
Linkage disequilibrium analysis results. Linkage disequilibrium was detected in rs3816769, rs744166 (D’ = 0.926, r2 = 0.768), and rs1053004 (D’ = 0.879, r2 = 0.704) (p < 0.001). Additionally, rs1053004 is in LD with rs744166 (D’ = 0.779, r2 = 0.0.596) and rs4796793 (D’ = 0.809, r2 = 0.378) (p < 0.001).

We examined genotype frequency patterns of linkage disequilibrium (LD) through classic approaches using r and D’ (Figure 2). The results show that rs3816769 is in LD with rs744166 and rs1053004 (p < 0.001). In addition, rs1053004 demonstrated LD with rs744166 and rs4796793 (p < 0.001). The r2 results are given in Table 5.

Multiple SNP analysis results led us to investigate haplotype frequencies in the patient and control groups (Tables 6 and 7). Therefore, we conducted a haplotype analysis using a minimum of 3% of occurrence in our sample. Notably, CCTT (line 1 in Table 6) was found to be the most prevalent haplotype in patients (60.5%) and healthy controls (58%). Furthermore, this study revealed substantial correlations between rare haplotypes and CAKUT (p = 0.041), as depicted in Table 7. The CCTC haplotype for rs744166, rs4796793, rs1053004, and rs3816769 polymorphism was exclusively observed in the CAKUT group. Conversely, the control group exclusively detected the CGTT haplotype for rs744166, rs4796793, rs1053004, and rs3816769 polymorphism.

## 4. Discussion

This study aimed to investigate four polymorphisms in the *STAT3* gene in patients with CAKUT. As a transcription factor, STAT3 is essential in differentiated tissue functions during vertebrate development, inflammation, immune control, and cancer. Variations in the *STAT3* gene are associated with autoimmune diseases and cancer [13,16]. The STAT3 pathway interacts with different signaling pathways and molecules, functioning as a central component of cell signaling systems. Studies have indicated that STAT3 activation contributes to ischemia-reperfusion injury and regulates proinflammatory cytokines. However, its role in nephritic pathology and sepsis-induced acute kidney injury (AKI) is still being investigated. While some studies have suggested that STAT3 exacerbates inflammation, others have proposed that it may help identify potential therapeutic targets. Further research and experiments are required in both cases. Maresin 1, an endogenous lipid mediator, has also been found to have antiinflammatory effects in attenuating sepsis acute kidney injury (SAKI). Although there are conflicting reports on the protective effects of early STAT activation in SAKI, the overall function of STAT3 in renal ischemia-reperfusion-induced AKI requires further investigation [17]. The association between the STAT3 pathway and LIFR function was previously reported, and the STAT3 pathway was considered an essential element of signaling in the urinary tract [12]. Our study investigated the functional polymorphisms of the *STAT3* gene since STAT3 inhibition affects urothelial inflammation [12].

Aghakhani et al. (2018) investigated the association between STAT3 polymorphisms associated with inflammation and susceptibility to cardiopulmonary bypass-related acute kidney injury (CPB-AKI). Their study analyzed the STAT3 rs1053004 and rs744166 polymorphisms in 129 patients. The rs1053004 SNP was reported to have effects on STAT3 expression. One study reported that STAT3 may play a role in AKI, and its expression is increased in tubular epithelial cells [8]. Another study on the Iranian population reported that AKI following CPB surgery decreased in individuals with the rs1053004 GG genotype. Additionally, rs1053004 AG genotype frequency was increased in patients with acute renal injury incidence after CPB surgery [18]. The position of rs1053004 in the 3’ untranslated region of the STAT3 gene suggests potential mediation through miR-423-5p and hsa-miR-99b-3p-region, biomolecules that play an important role in gene regulation. This finding may be linked to the dual role of STAT3 in regulating inflammatory and antiinflammatory responses, influencing T cell differentiation and function. Aghakhani et al. (2018) suggest that the G allele of rs1053004 may affect miRNA binding, leading to increased STAT3 expression and the creation of an antiinflammatory environment, consequently reducing the risk of CPB-AKI in individuals with the GG genotype. However, the frequency of rs744166 polymorphism was almost the same in the patient and control groups. Aghakhani et al. (2018) did not observe a significant association between the rs744116 variant and CPB-AKI.

We analyzed genotype frequencies and OR of the examined SNPs in both the patient and control groups. Our study reveals no statistically significant difference in the genotype frequencies of the SNPs between CAKUT patients and control individuals when they were analyzed individually (Table 3). However, the results show that individuals with the TT for rs1053004 have a 1.23 (0.53–2.90, OR 95% CI) times higher risk of developing CAKUT than those with the CC allele. Additionally, patients with the CC allele for rs3816769 are 1.41 (0.52–3.83, OR 95% CI) times more likely to develop CAKUT than those with the TT allele. These findings suggest that rs1053004 and rs3816769 polymorphisms may contribute to the development of CAKUT (Table 3).

We analyzed LD for the SNPs and evaluated the frequency of coinheritance of alleles. LD analysis is a powerful tool in population genetics and provides data related to the nonrandom association of alleles at various loci within a population. This analysis yields valuable insights into populations’ genetic structure and evolutionary history [19]. D’ and r2 values indicate the closeness of the two loci and their coinheritance (Tables 5–7). A positive D’ value means that the gamete is more frequent than expected, while a negative value indicates that the combination of these two alleles is less frequent than expected. We observed that rs3816769 is in LD with rs744166 (D’ = 0.926, r2 = 0.768) and rs1053004 (D’ = 0.879, r2 = 0.704) (p < 0.001). Furthermore, rs1053004 is in LD with rs744166 (D’ = 0.779, r2 = 0.596) and rs4796793 (D’ = 0.809, r2 = 0.378) (p < 0.001) (Figure 2). In addition to these statistical analyses, a haplotyping analysis was performed, and the CCTT haplotype was found to be the most prevalent haplotype in patients (60.5%) and controls (58%) (Table 6). Moreover, some rare haplotypes were significantly higher (p = 0.041) in CAKUT patients than in healthy controls. The CGTT haplotype was exclusively observed in the control group, and individuals with this haplotype were not detected in the patient cohort. Furthermore, the TCTC haplotype was five times more prevalent in the control group than in the patient group, whereas the GCCC haplotype was six times more common in the control group. The present study suggests that the CGTT, TCTC, and GCCC haplotypes may have a protective effect on CAKUT. However, the CCTC haplotype was identified exclusively in patients and was not observed in the healthy control group, suggesting that the CCTC haplotype may increase susceptibility to CAKUT (Tables 6 and 7). Many of these nonrandom combinations can be clinically associated with CAKUT. The findings reveal a compelling possibility that specific uncommon haplotypes are more frequently observed in individuals suffering from CAKUT. Identifying these haplotype blocks could lead to a better understanding of the genetic factors contributing to the development of CAKUT, which could facilitate the development of more effective treatment and therapy to manage or prevent CAKUT and its associated symptoms.

The study has limitations. The first limitation is the study’s sample size. Researching CAKUT is challenging due to the diverse patient subgroups and limited number of patients and controls. We included more patients than the sample size determined by power analysis to address this issue. The second limitation is the inadequate number of patients in the subgroups. It is challenging to find patients with well-defined phenotypes to place into specific subgroups because some exhibit phenotypes that overlap with different subgroups. Including more patients in the subgroups would be beneficial in identifying genetic factors related to specific CAKUT subgroups. The third limitation is the presence of a larger number of potential major genes and modifying genes in the genetic background of the populations, which play a crucial role in the development of the CAKUT phenotypes. Although genetic background may vary between populations, leading to differing frequencies of SNPs, this does not impact the relationship between the STAT3 gene and the CAKUT phenotype. It is important to conduct functional studies to fully evaluate and substantiate this study’s positive outcomes.

## 5. Conclusion

This is the first study investigating the effects of STAT3 functional polymorphisms among CAKUT patients. The majority of the STAT3 activation was reported to be associated with renal diseases in humans, including diabetic nephropathy and acute renal injury, as well as chronic renal disorders. According to the research data, a persuasive possibility exists that specific atypical haplotypes are more frequently observed in individuals suffering from CAKUT. We also found that the TT genotype for rs1053004 increases CAKUT risk by 1.23-fold, while the CC genotype for rs3816769 raises the risk by 1.41-fold. These gene variations may contribute to CAKUT development.

Renal failure typically results from genetic and environmental factors affecting gene expression in kidney tissue. In individuals with CAKUT, developmental abnormalities can lead to chronic kidney disease and, eventually, kidney failure. Further research is needed to investigate differences in gene expression patterns and signaling mechanisms between clinically homogeneous subgroups of CAKUT patients. These studies could provide deeper insights into the pathophysiology of the disease and facilitate the development of personalized treatment approaches.

## Figures and Tables

**Table 1:** Primer sequences and restriction endonuclease enzymes used for analyzing rs4796793 and rs744166 SNPs. bps: base pairs

SNP	Forward Primer (5’ – 3’)	Reverse Primer(5’ – 3’)	Amplicon length (bps)	Restriction Endonuclease
**rs4796793**	TCTGGTAGACACAGCTCAGTATGG	CCATAGTCGCAGAGGTAGATTTTA	502	MwoI
**rs744166**	TCAGCTGGAGTACAAACCCTG	TTACAGAGCTACATGTGATGGGA	329	AluI

**Table 2:** Demographic and clinical features of the study group.

	Patients		Controls
**Median Age (month) (min-max)**	76.4 (1–216)		73.2 (6–216)
**Male/Female**	75/70		68 / 60
**Symptoms of the patients’**	**n (%)**	**Sex (Male / Female) (n)**	**The median age of diagnosis (months) (min-max)**
**Hydronephrosis**	32 (22.1%)	24/8	25.3 (1–156)
**Vesicoureteral Reflux**	41 (28.3%)	14/27	51.4 (1–216)
**Chronic Kidney Disease**	21 (14.4%)	15/6	94.8 (2–204)
**Renal hypoplasia and Agenesis**	20 (13.8%)	13/7	73.4 (1–192)
**Urinary Tract Infections**	31 (21.4%)	9/22	55.9 (1 216)

**Table 3:** Distribution of genotype and allele frequencies of rs1053004, rs3816769, rs4796793, and rs744166 in CAKUT patients and healthy controls.

SNP	Genotype	Patient	Control	OR (95% CI)	P-value
**rs1053004**	CC	13 (9%)	13 (10.2%)	Reference	0.816
	CT	70 (48.3%)	57 (44.5%)	1.07 (0.46–2.50)	
	TT	62 (42.8%)	58 (45.3%)	1.23 (0.53–2.90)	
	C	96 (33%)	83 (32%)	Reference	0.866
	T	194 (67%)	173 (68%)	0.97 (0.68–1.39)	
**rs3816769**	TT	65 (44.8%)	65 (50.8%)	Reference	0.494
	TC	68 (46.9%)	56 (43.8%)	0.82 (0.50–1.35)	
	CC	12 (8.3%)	7 (5.5%)	1.41 (0.52–3.83)	
	T	201 (69%)	177 (69%)	Reference	0.966
	C	89 (31%)	79 ((31%))	0.99 (0.69–1.40)	
**rs4796793**	CC	92 (63.5%)	72 (56.2%)	Reference	0.441
	GC	47 (32.4%)	51 (39.8%)	0.721 (0.44–1.19)	
	GG	6 (4.1%)	5 (3.9%)	0.939 (0.28–3.20)	
	C	231 (80%)	195 (76%)	Reference	0.327
	G	59 (20%)	61 (24%)	0.816 (0.54–1.22)	
**rs744166**	TT	15 (10.3%)	11 (8.6%)	Reference	0.789
	TC	66 (45.5%)	63 (49.2%)	0.768 (0.33–1.80)	
	CC	64 (44.1%)	54 (42.2%)	0.869 (0.37–2.05)	
	T	96 (33%)	85 (33%)	Reference	0.980
	C	194 (67%)	171 (67%)	1.00 (0.70–1.43)	

* Logistic regression analysis was used to obtain ORs and confidence intervals.

**Table 4:** Distribution of genotype frequencies in the patient and control groups to show whether they are consistent with Hardy–Weinberg equilibrium; >0.05 indicates consistency with Hardy–Weinberg equilibrium.

SNPs	Control	Patient
**rs1053004**	0.85	0.28
**rs3816769**	0.03	0.52
**rs744166**	0.22	0.74
**rs4796793**	0.27	0.99

* Chi-square goodness of fit was used to test Hardy–Weinberg equilibrium.

**Table 5:** r2 statistic results for linkage disequilibrium.

	rs744166	rs4796793	rs1053004	rs3816769
rs744166	-	0.413063	0.596138	0.767726
rs4796793	-	-	0.378348	0.471557
rs1053004	-	-	-	0.703753
rs3816769	-	-	-	-

**Table 6:** Haplotype frequency estimations (n = 273).

	rs744166	rs4796793	rs1053004	rs3816769	Total	Patient	Control	Cumulative frequency
1	C	C	T	T	0.594	0.605	0.580	0.594
2	T	G	C	C	0.191	0.196	0.184	0.785
3	T	C	C	C	0.084	0.082	0.086	0.869
4	C	C	C	T	0.038	0.045	0.031	0.908
5	T	C	T	T	0.034	0.037	0.031	0.942
6	C	G	T	T	0.018	NA	0.039	0.961
7	T	C	T	C	0.008	0.003	0.015	0.970
8	T	G	T	C	0.008	0.007	0.009	0.978
9	C	C	T	C	0.008	0.014	NA	0.986
10	C	C	C	C	0.007	0.002	0.012	0.993

* EM algorithm used to estimate haplotype frequencies

**Table 7:** Haplotypes in patients with CAKUT.

	rs744166	rs4796793	rs1053004	rs3816769	Freq	OR (95% CI)	P-value
**1**	C	C	T	T	0.593	Reference	
**2**	T	G	C	C	0.191	1.01 (0.63–1.62)	0.96
**3**	T	C	C	C	0.084	1.10 (0.59–2.04)	0.77
**4**	C	C	C	T	0.039	0.70 (0.27–1.79)	0.45
**5**	T	C	T	T	0.035	0.89 (0.36–2.23)	0.81
**rare**	*	*	*	*	0.056	2.29 (1.04–5.06)	0.041
Global haplotype association p = 0.34							

* Logistic regression analysis conducted to obtain ORs

## Data Availability

The datasets generated and analyzed during the study are available from the corresponding author upon reasonable request.
